# Diffusion Kurtosis Imaging of Neonatal Spinal Cord in Clinical Routine

**DOI:** 10.3389/fradi.2022.794981

**Published:** 2022-05-23

**Authors:** Rosella Trò, Monica Roascio, Domenico Tortora, Mariasavina Severino, Andrea Rossi, Julien Cohen-Adad, Marco Massimo Fato, Gabriele Arnulfo

**Affiliations:** ^1^Departments of Informatics, Bioengineering, Robotics, and System Engineering, University of Genoa, Genoa, Italy; ^2^Neuroradiology Unit, Istituto Giannina Gaslini, Genoa, Italy; ^3^Department of Health Sciences, University of Genoa, Genoa, Italy; ^4^NeuroPoly Lab, Institute of Biomedical Engineering, Polytechnique Montreal, Montreal, QC, Canada; ^5^Functional Neuroimaging Unit, CRIUGM, Université de Montréal, Montreal, QC, Canada; ^6^Mila—Quebec AI Institute, Montreal, QC, Canada; ^7^Neuroscience Center, Helsinki Institute of Life Science, University of Helsinki, Helsinki, Finland

**Keywords:** spinal cord, diffusion tensor imaging (DTI), diffusion kurtosis imaging (DKI), image processing pipeline, neonatal imaging, punctate white matter lesions

## Abstract

Diffusion kurtosis imaging (DKI) has undisputed advantages over the more classical diffusion magnetic resonance imaging (dMRI) as witnessed by the fast-increasing number of clinical applications and software packages widely adopted in brain imaging. However, in the neonatal setting, DKI is still largely underutilized, in particular in spinal cord (SC) imaging, because of its inherently demanding technological requirements. Due to its extreme sensitivity to non-Gaussian diffusion, DKI proves particularly suitable for detecting complex, subtle, fast microstructural changes occurring in this area at this early and critical stage of development, which are not identifiable with only DTI. Given the multiplicity of congenital anomalies of the spinal canal, their crucial effect on later developmental outcome, and the close interconnection between the SC region and the brain above, managing to apply such a method to the neonatal cohort becomes of utmost importance. This study will (i) mention current methodological challenges associated with the application of advanced dMRI methods, like DKI, in early infancy, (ii) illustrate the first semi-automated pipeline built on Spinal Cord Toolbox for handling the DKI data of neonatal SC, from acquisition setting to estimation of diffusion measures, through accurate adjustment of processing algorithms customized for adult SC, and (iii) present results of its application in a pilot clinical case study. With the proposed pipeline, we preliminarily show that DKI is more sensitive than DTI-related measures to alterations caused by brain white matter injuries in the underlying cervical SC.

## Introduction

In recent years, an increasing number of works in the field of neuroimaging are stressing the importance to move beyond the simplistic assumptions of diffusion tensor imaging (DTI) model (1) toward more advanced diffusion MRI (dMRI) methods, among which diffusion kurtosis imaging (DKI) (2) is one of the most promising (3).

Within existing non-standard techniques, DKI has indeed turned out to be especially suitable for imaging of the spinal cord (SC), a structure where the assumption of Gaussian diffusion fails (4). Indeed gray matter (GM) in the central portion of the SC contains cell membranes and organelles that limit diffusion to fewer directions. Taking into account the pathological processes not following Gaussian distribution, DKI provides a better understanding of the underlying micromolecular environment. In fact, it exhibits increased sensitivity in microstructural assessment of both white matter (WM) and GM (5). Hence, this susceptibility translates into an increased amount of diagnostic information beyond that obtained with routine diffusion metrics, as proven both for adult brain (2, 5) and spine (6, 7).

Latest technological advances on reduced field-of-view techniques to mitigate susceptibility artifacts and cardiac/respiratory gating have allowed experts to overcome most of the methodological challenges inherent to adult SC imaging (8). Thanks to these strategies, DKI by now represents a promising tool for studying a plethora of spine disorders, with minor modifications to protocol parameters in use for brain imaging (6, 9–12).

The scenario becomes definitely more complicated when attempting to translate this imaging technique to the pediatric clinical setting (13). Typical issues inherent to the SC district include a small cross-sectional area requiring high spatial resolution, interface between regions with different magnetic properties, partial volume effect (PVE) of pulsating cerebro-spinal fluid (CSF) with each heartbeat, and bulk physiologic motion due to the proximity of the heart and the pulmonary parenchyma. This scenario is further complicated here from a multiplicity of factors related to the age range under analysis. Children in general have smaller anatomical structures—which, in turn, might result in a higher risk of radiofrequency heating effects—and move more frequently (e.g., tongue sucking motion). On the other hand, artifact-reducing techniques (i.e., cardiac gating, respiratory compensation, and suppression sequences) (14) are often unfeasible since they are time-consuming, and sedation is typically not desirable. All the aforementioned issues result in artifact-laden, low-signal images, which are often suboptimal for diagnostic evaluation.

The adopted solutions for improving image resolution and reducing artifacts comprise induction of natural sleep (by feeding the patient immediately before MR examination), the use of a vacuum fixation pillow to wrap the patient, and the use of special earmuffs to protect from noise. However, the main requirement when handling with pediatric dMRI data is the choice of a proper acquisition protocol tailored for pediatric imaging, which is made up of low angular resolution, low *b*-values, and few gradient directions, likewise in pediatric brain in order to minimize scan time. Nevertheless, this forced time minimization clashes with specific requirements of advanced diffusion methods in terms of acquisition sequences.

Contrary to DTI, indeed DKI, as with all higher-order diffusion models, requires multi-shell high-angular-resolution diffusion imaging (HARDI) sequences (15), typically involving at least three non-zero *b*-values distributed on hundreds of gradient directions, grouped in shells. This implies longer acquisition time, straining the feasibility of advanced dMRI methods in pediatrics. Resorting to optimized acquisition sequences (16), often combined with state-of-the-art techniques such as parallel imaging (17) and multi-band, can significantly increase the acquisition speed and reduce the artifacts. However, these advanced technologies are not always available in a general hospital due to high costs and technical limitations.

If extension of DTI to the pediatric SC has shown promising results in a wide range of clinical conditions, as evidenced by the increasing number of works on the topic (18–24), what immediately stands out while reviewing the literature on pediatric SC is the absence of studies concerning DKI and particularly applied to the neonatal period (0 to 1 month).

To the best of our knowledge, the only published work on pediatric DKI (25) is limited to grown-up children (6–16 years), whose larger anatomical structures and reduced source of movements enable better image quality and longer scan times. In newborns, indeed the SC dimensions themselves–24-cm average length and 4.4-mm diameter, possibly further decreasing in case of malformations (26) are enough to conceive amplification of the aforementioned technical issues and thus justify the lack of research toward this direction.

However, the ability of DKI to offer additional and complementary information to DTI may bring a significant contribution in investigating such decisive and delicate stage of development, especially if we consider the wide range of developmental anomalies of the spinal canal affecting infants at birth (27).

It is on this premise that we conceived our work, the aim of which is to show the feasibility of applying DKI to neonatal SC within clinical routine with all the issues that this entails, opting for minimal modifications of the current clinical setup.

We thus introduce here the first complete pipeline specifically adapted to neonatal imaging acquired for diagnostic purposes. The applicability and clinical validity of the proposed method have been evaluated, by analyzing a specific clinical case study concerning a condition common to preterm birth, in collaboration with the Neuroradiology Unit of Giannina Gaslini Children's Hospital of Genova.

Specifically, we assessed the effects of WM brain lesions typical of periventricular white matter injury (PWMI) on lower cervical SC tracts by comparing the diffusion measures between pathological patients and healthy controls. Our findings, though preliminary, confirm the ability of the DKI model in capturing subtle pathological alterations. Conversely, DKI-related measures appear to be less sensitive to WM/GM tissue differentiation at this stage.

Since there are currently neither available protocols nor standardized methodological pipelines for performing DKI in the infant SC, this methodological outline may, at least, serve as a proof-of-concept, stressing the need for infant-specific data acquisition and processing guidelines in order to translate the DKI of neonatal SC into routine clinical practice.

## Materials and Methods

### Subjects

Infants whose data have been used to disclose each step of the pipeline have been enrolled since August 2019 and scanned with 3.0-T MR scanner using a 32-channel head array coil (Ingenia Cx, Philips, Best, The Netherlands) at the Neuroradiology Unit of Giannina Gaslini Children's Hospital of Genova. Conventional MRI and DKI were performed in 17 pre-term infants [28.1–36.7 weeks gestational age (GA); scanned at term-equivalent age (TEA)]. Diagnosis has been exclusively made based on MRI findings as reported by experienced neuroradiologists. Details about the subjects' demographics are reported in Table 1.

**Table 1:** Demographic features of infants.

	**Unhealthy** **(*n* = 9)**	**Healthy** **(*n* = 8)**
Gender (M/F)	6/3	4/4
Mean GA (range; week)	30.3 ± 2.6 (28.1–35.0)	31.8 ± 3.1 (28.3–36.7)
Mean PNA (range; week)	9.2 ± 3.9 (0.1–11.7)	8.6 ± 3.6 (2.0–10.7)
Mean PMA (range; week)	39.4 ± 1.6 (35.1–40.6)	40.4 ± 1.3 (38.7–42.4)

*M/F, number of male and female infants; GA, gestational age; PNA, postnatal age; PMA, postmenstrual age*.

This single-center study was carried out in accordance with the recommendations of “Comitato Etico Regione Liguria, Genova, Italy”, with written informed parental consent obtained for each infant prior to examination in accordance with the Declaration of Helsinki. The subjects were spontaneously breathing during the examination; free-flowing oxygen was administered for the whole duration of the MRI session if necessary. Throughout the course of the examination, the newborns were subjected to constant monitoring of oxygen saturation and heart rate by a pulse oximeter and a three-electrode electrocardiographic monitor, respectively.

In consensus with a board-certified pediatric neuroradiologist, we performed quality control (QC) for each of the pipeline's steps.

### Full Pipeline Description

Our pipeline integrates MRtrix3 (v.3.0.1) (28) for setting of the dMRI acquisition sequence, Spinal Cord Toolbox (SCT, v. 5.3.0, https://github.com/neuropoly/spinalcordtoolbox) (29) for all processing steps specific to the SC, and Diffusion Imaging in Python (Dipy, v.1.4.0) (30) for denoising as well as computation of diffusion metrics.

The output of key processes, such as motion correction, segmentation, and registration with atlas, can be checked through an interactive SCT QC module, which automatically generates reports consisting of HTML files and containing a table of entries which allow to show, for each entry, animated images (background with overlay on and off) for data quality validation.

In our methodological pipeline, we have opted to mainly rely on SCT, being currently the only existing fully comprehensive, free, and open-source software dedicated to the processing and analysis of multi-parametric MRI of the spinal cord successfully employed in a plethora of clinical applications concerning adult SC (31–44).

**Figure 1:**
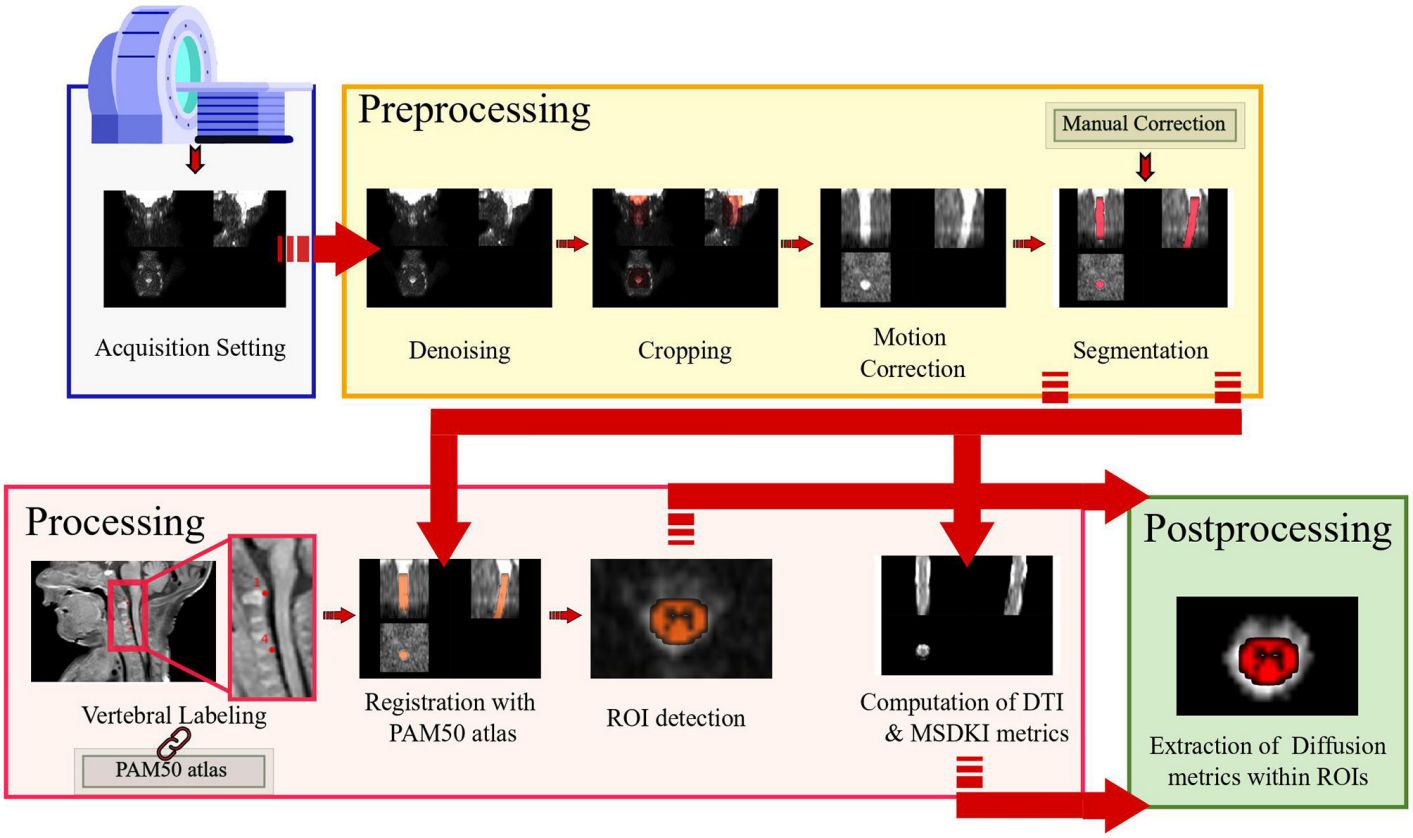
Overall processing pipeline: the designed pipeline allows complete handling of diffusion kurtosis imaging scan of neonatal spinal cord from acquisition setup to preprocessing, processing, and postprocessing.

An overview of our image processing pipeline highlighting the key features is shown in Figure 1. Since SCT algorithms are validated in adult imaging, we specifically customized each processing step to our neonatal scans. Our pipeline thus represents, to the best of our knowledge, the first semi-automated *ad-hoc* procedure for imaging of neonatal spine. A fully automatic workflow is not feasible here—acquisition time constraints, available scanner features, and subsequent image quality require inevitable, although minimal and highly reproducible, manual interventions.

### Customized Acquisition Setting

In order to minimize macroscopic movement artifacts, all recommended guidelines for pediatric imaging have been adopted. So as to protect infants from acoustic disturbances caused by MR sequences, we resorted to baby earmuffs and silicone paste for hearing aids. Furthermore, we avoided most of the motion by swaddling the infants and by placing airbags around their head. In addition, protective pads have been placed between the magnet and the patient. All these contribute to create a comfortable and warm rest environment, thus minimizing the chance of free movements.

MRI was performed when possible during spontaneous sleep by exploiting the administration of breast milk or formula about 30 min before the start of the exam. In case of spontaneous sleep failure, in order to minimize macroscopic movement artifacts, the instrumental examination was performed under mild sedation by orally administering midazolam at 0.1 to 0.2 mg/kg diluted in 33% glucose solution, subject to the signature of informed consent from parents and applied by expertly trained nurses.

Given the lack of a specific acquisition protocol for DKI of neonatal SC, we designed the diffusion-weighting scheme in collaboration with the neuroradiologists at Giannina Gaslini Hospital. One constraint we had to deal with was the impossibility to perform optimized variants of spin-echo echo planar imaging (SE-EPI) sequence [i.e., reduced field-of-view (FOV) or spatially selective techniques] (16) on a Philips Ingenia scanner. Therefore, minimization of scan duration was our main focus in order to suppress motion and fast CSF pulsation artifacts typical of newborns.

We thus tested different versions of diffusion-weighted gradient scheme, adopting an optimal tradeoff between fiber orientation distributions profile (estimated with Mrtrix3 using multi-shell multi-tissue constrained spherical deconvolution), image quality, and scan time.

We generated each multi-shell diffusion gradient table through Mrtrix3 script *gen_scheme*, taking as inputs the number of phase-encoding directions to be included in the scheme (for most scanners, including ours, typically 1), the *b*-value of the shell, and the number of directions to include in the shell. This procedure ensures uniform spherical sampling by maximizing uniformity within shells using a bipolar electrostatic repulsion model for optimal angular coverage.

As regards the choice of acquisition parameters, we borrowed some crucial measures (*b*-values, voxel size, as well as TR/TE) from the setting used in the corresponding adult study that we referred to as a starting point (45). Indeed this group presented a scenario closely similar to ours—Philips 3T scanner and SE-EPI sequence without advanced variants—and managed to perform DKI in adult subjects within a clinically feasible time period, e.g., 6 min.

For further reducing the acquisition time without significantly affecting the image quality, we applied the MultiBand slice acceleration technique (46) (https://www.usa.philips.com/healthcare/resources/landing/the-next-mr-wave/compressed-sense).

**Figure 2:**
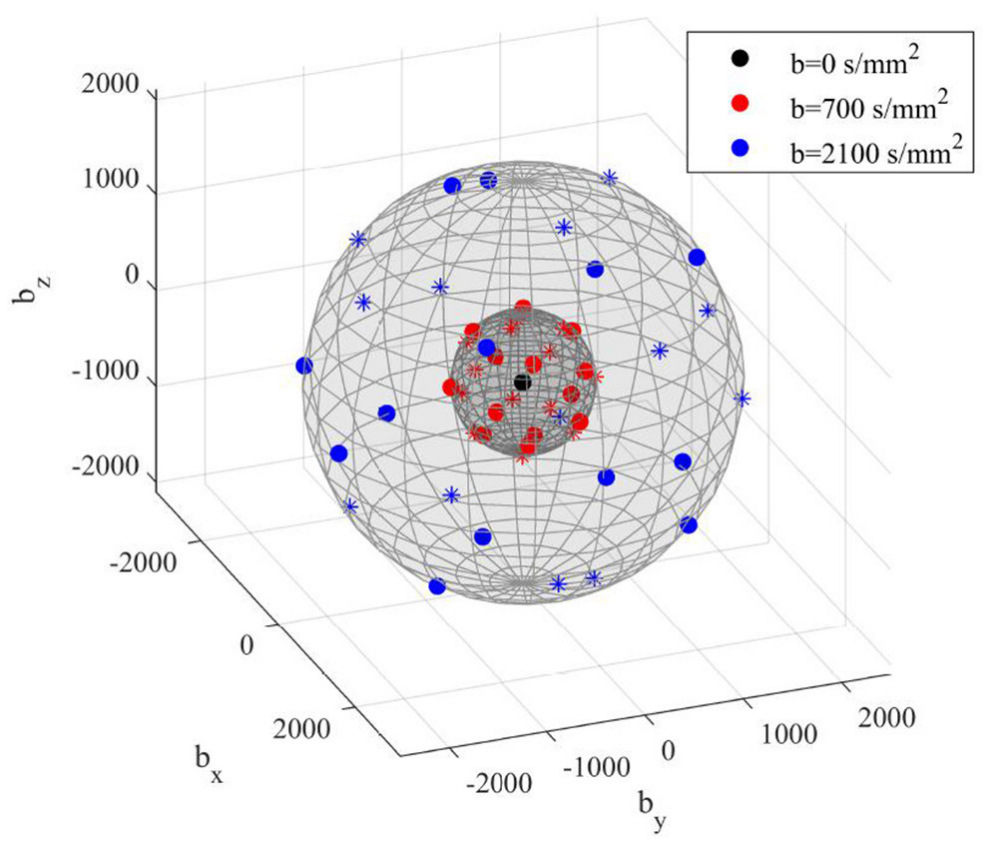
3D view of final diffusion acquisition scheme: directions of diffusion-sensitizing gradients relative to each *b*-value are displayed in three different colors as reported in the legend. Units are in s/mm^2^. Markers indicate polarity: dots are the polarities in the set; asterisks are their opposite.

The final version of the diffusion acquisition scheme is displayed in Figure 2 as well as reported in Table 2 and includes 6 *b* = 0, 13 *b* = 700, and 13 *b* = 2,100 s/mm^2^ for a duration of 4 min and 30 s. This allowed the acquisition of high-in-plane-resolution axial-diffusion-weighted images, where *b* = 0 scans could be well discriminated from non *b* = 0 volumes and the anatomical SC features are sharp.

**Table 2:** Data acquisition details for both structural 3D T1w and DKI image.

	**3dT1**	**DKI**
TR/TE (s)	0.6/0.026337	3.378/0.128
Diffusion scheme (s/mm^2^)	–	6 *b* = 0, 13 *b* = 700, 13 *b* = 2100
Flip angle (°)	90	90
Field-of-view (mm)	195 × 195 × 126	128 × 93 × 96
In-plane acquisition resolution (mm)	1^*^1	1^*^1
Acquisition matrix	195^*^195	128^*^93
In-plane reconstruction resolution (mm)	0.38^*^0.38	0.8^*^0.8
Reconstruction matrix	512^*^512	160^*^160
Multi-band factor	–	2
Averages	2	1
Slice thickness (mm)	0.5 without gap	4, without gap
Slice orientation	Sagittal	Axial
Slices	251	24
Total scan time	4 min, 5 s	4 min, 30 s
Partial fourier factor	–	0.6

A valuable alternative to this reduced DKI scheme is represented by fast kurtosis imaging (47). This recently developed technique may give a relevant boost to the widespread, routine clinical applicability of DKI in the infant SC by drastically reducing the acquisition as well as post-processing time.

A significant increase in speed is indeed made possible by a reduction in data demand achieved through the rigorous analysis of the relation between the DKI signal and the kurtosis tensor-based metrics. It therefore computes a mean of the kurtosis tensor from at least 13 diffusion-weighted images (dwis)—the so-called 1-3-9 approach. Commonly, this basic scheme is extended to 19 diffusion-weighted images—the so called 1-9-9 approach—for robust and reliable parameter estimation with the chance to reconstruct some parameters even in real time, which may be really valuable in the clinic.

This technique has been successfully validated both in human brain (48, 49) and spinal cord (50), demonstrating to offer the same information as the conventional DKI both in normal and diseased tissue.

Moreover, under the assumption of axisymmetry inherent to regions with a well-defined axis of symmetry, such as the large peripheral nerves and spinal cord (51), this method can also be easily integrated with white matter tract integrity. This valuable modeling-based WM characterization (52) provides detailed information about the microstructure of highly aligned fiber bundles and could thus be particularly suitable for investigating SC.

Both “1-3-9” and “1-9-9” methods are heavily sensitive to deviations from the encoding scheme required to ensure data reduction. These schemes consist in acquiring images at fixed *b*-values (0, 1,000, and 2,500) along a precise set of directions specified in Hansen et al. (53).

This is thus about conventional diffusion sequences easily implemented on almost any clinical system by allowing the inclusion of DKI, at a little additional cost, as a component of any protocol for imaging of the brain or other organs. However, our starting acquisition scheme did not match the required diffusion-sensitizing directions, and exactly for this reason, we were not able to apply this method retrospectively.

Nonetheless, a strength of the current methodological pipeline is its independence from the dMRI acquisition scheme used to acquire input raw data, and it could thus be successfully used to perform fast DKI, too.

Along with dMRI, we also acquired a high-resolution structural image as an anatomical reference. The definitive MRI protocol thus consisted in a turbo spin echo 3D T1-weighted image followed by a DKI series whose details are listed in Table 2.

### Preprocessing

#### Denoising

SC imaging is characterized by low SNR, which can hamper accurate, repeatable, quantitative measurements. Moreover, models such as DKI are susceptible to noise and signal fluctuations, often leading to degeneracies in the estimation of derived parameters. SNR further lowers, in the case of neonates, due to the relatively high overall free water content, and denoising approaches based on principal component analysis (PCA) are inapplicable due to a reduced number of diffusion gradient directions.

Therefore, we adopted Patch2Self, a recently proposed self-supervised learning denoising method that outperforms existing non-supervised methods (54).

A unique advantage of Patch2Self is the lack of requirement for selecting or calibrating an explicit model either for noise or diffusion signal so that it can be applied at any step in the pre-processing pipeline. The only assumption it relies on is randomness and uncorrelation of noise across different gradient directions. Its framework consists in holding out one volume and using patches from all other volumes to predict the center of the patches of the held-out volume using a regressor. This denoiser has already showed a significant improvement in repeatability and conspicuity of pathology in diffusion volumes and quantitative DTI metrics for adult SC (55).

**Figure 3:**
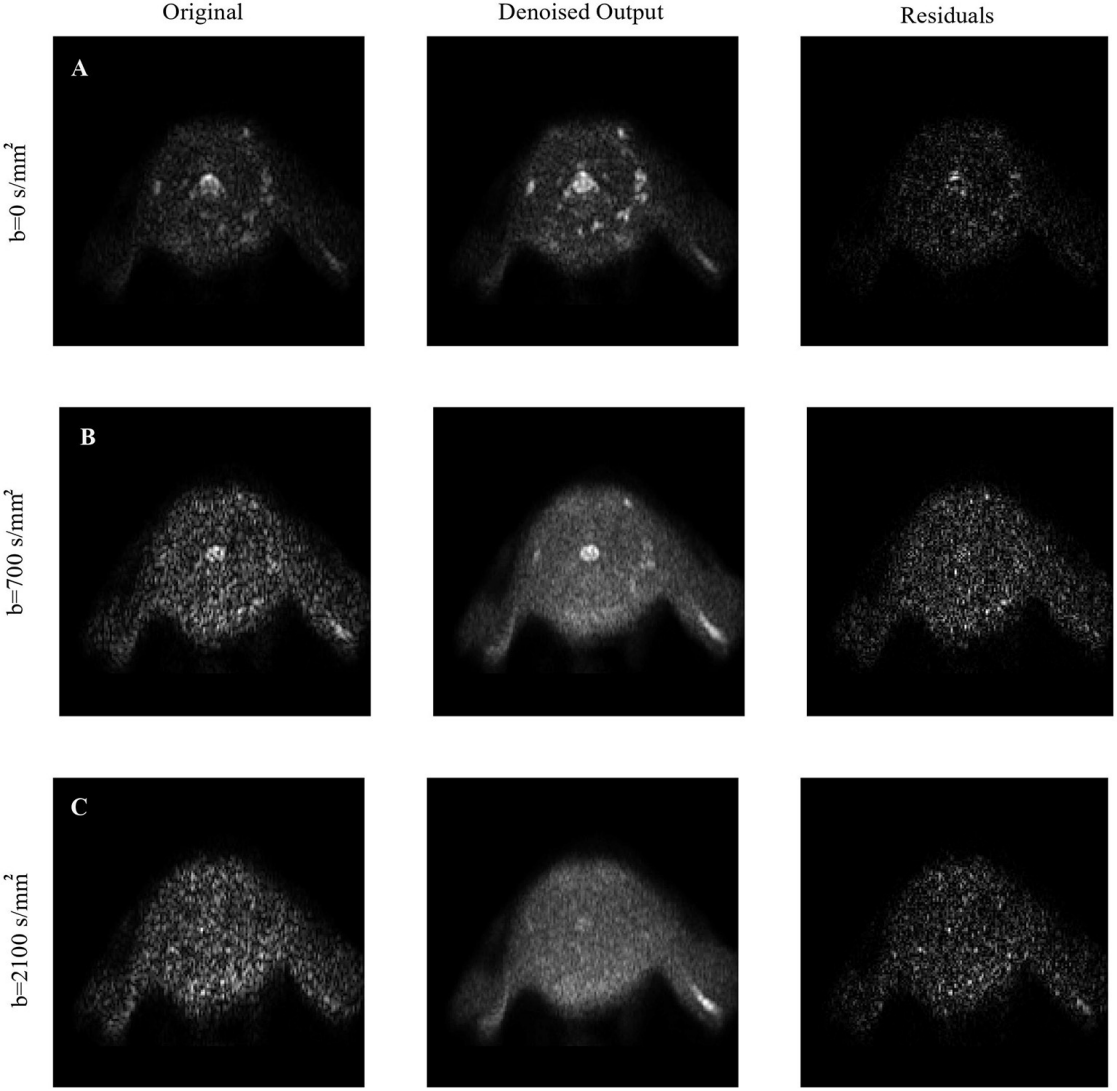
Visual inspection of denoising: The denoising of Patch2Self is compared against the original noisy image along with their corresponding residuals for each **(A)** *b* = 0, **(B)** *b* = 700, and **(C)** *b* = 2,100 s/mm^2^ shells, respectively. Notice that Patch2Self does not show any anatomical structure in the corresponding residual plots and likely neither introducing structural artifacts.

Here we chose to apply Patch2Self as the first preprocessing step on raw data since it showed to offer the highest SNR. The method is implemented in Dipy v.1.4.0 and applied with ordinary least squares regressor as recommended for SC imaging (Figure 3).

#### Cropping

SC scans also usually include cerebral areas, such as medulla and cerebellum, due to their proximity with cervical SC (cSC). In order to exclusively focus on the area of interest excluding undesired voxels, as a first preprocessing step, we thus recommend applying to DKI images the SCT function *sct_crop_image*, allowing also to fasten subsequent processing. Lower and higher bounds for cropping along the three spatial coordinates can be specified *via* command line in order to select the same area of interest (i.e., cSC) for all the cohorts, considering that FOV positioning is consistent across subjects.

**Figure 4:**
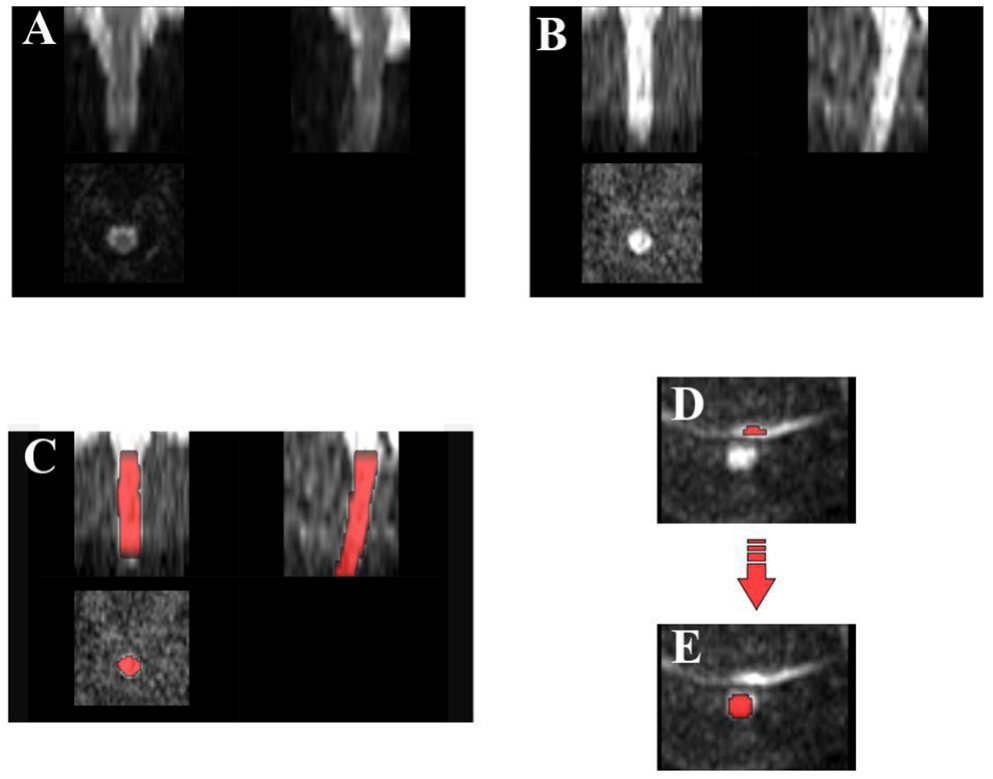
Preprocessing: diffusion kurtosis imaging scan through preprocessing steps for one example subject: **(A)** field-of-view reduction, **(B)** motion correction, **(C)** segmentation: deep learning segmentation algorithm generally achieves satisfactory results in spinal cord detection, **(D)** example of artifactual slice due to poor fat saturation, causing the fat to alias on the spinal cord area, and **(E)** requiring manual correction of segmentation.

Specifically, in the case of our scans, FOV reduction allowed to exclude upper non-spinal areas (i.e., cerebellum) as well as lower spinal levels whose corresponding slices are not usable due to poor image quality (Figure 4A).

#### Motion Correction

The subjects' immobilization and anesthesia successfully minimized motion in our acquisitions. However, since dMRI data are analyzed at the voxel level, residual intrascan and/or interslice motion can adversely affect the accuracy of the modeled results. We thus resorted to SCT complex motion correction framework *sct_dmri_moco* based on a combination of tools.

First of all, *SliceReg* algorithm estimates slice-by-slice translations while ensuring regularization constraints along the z-axis. The latter is achieved using a polynomial function (order specified by the user, flag *-param*). This method was shown to offer better accuracy and robustness than rigid-body transformations and non-regularized slice-by-slice registration, respectively (29).

Moreover, motion correction in SCT includes another feature first proposed in Xu et al. (56) to improve the robustness of registration in high-*b*-value diffusion MRI data such as DKI datasets. It consists in grouping adjacent volumes and estimating the transformation relying on these successive subsets (typically from 3 to 5 volumes) averaged together (flag *-g*).

This robust slice- and group-wise motion correction works successfully also in the case of neonatal scans, and it is hence applied here with default parameters: grouping of 3 successive dMRI volumes, regularization with 2^nd^-order polynomial function, unitary smoothing kernel (1 mm), and final spline interpolation (flag -x), except for the metric used for registration (Figure 4B). Indeed cross-correlation (CC) has been selected as a similarity metric given its better performance with respect to mean squares or mutual information (default option) at the expense of computational time.

Since *sct_dmri_moco* works through iterative average over groups of successive slices in order to increase the SNR of the target image, its output includes a 3D volume corresponding to the mean from DKI slices. These motion-corrected average DKI data will serve as input for subsequent segmentation, thanks to its excellent cord contrast.

Thanks to the limited duration of our acquisition and to the adopted procedures for minimizing movement throughout the exam, the amount of motion is very limited in our images. As a result, the outcome of motion correction step does not significantly differ from a raw DKI image by visual assessment. However, this represents a crucial step in the case of longer scans that are more prone to source of motion artifacts.

#### Segmentation

Proper segmentation of SC is decisive for the subsequent steps of template registration and computation of metrics along the cord.

Detection of SC has turned out to be a critical step since the standard SCT algorithm *propseg*, based on multi-resolution propagation of tubular deformable models (57), is trained for adult spine.

Given the reduced size of neonatal SC and the low contrast between the spine and CSF, default segmentation method fails in several slices even after modulating the algorithm parameters—e.g., manual initialization of spinal cord centerline through interactive viewer (flag *-init-mask*), selection of SC radius size (flag *-radius*), or cord rescale (flag *-rescale*).

We thus resort to a more recent and advanced method of SC extraction based on deep learning *sct_deepseg_sc* (58). This fully automatic segmentation framework was conceived for detecting SC and intramedullary MS lesions from a variety of MRI contrasts and resolutions.

It is composed of a cascade of two convolutional neural networks specifically designed to deal with spinal cord morphometry: the first detects the cord centerline and reduces the space around the spinal cord (for better class balance), and the second segments the cord.

The segmentation results outperformed *sct_propseg*, showing higher robustness to variability in both image parameters and clinical conditions.

Thanks to its versatility, the application of this method come sout suitable also for neonatal imaging, allowing the robust and accurate segmentation of our scans without the ever need of additional parameters except just specifying the kind of image contrast as *dwi* (flag *-c*) (Figure 4C).

In case of failure of SC detection, we necessarily opt for manual correction of problematic slices on FSL editor (*FSLeyes*) (Figures 4D,E).

This is the case of five subjects within our cohort: to validate the quality of segmentation, we checked the QC feature on our MRI images across subjects and noticed some local segmentation leakage—related to the onset of artifacts at the acquisition phase and not to a flaw with the algorithm—in a few slices and hence corrected it manually.

### Processing

#### Vertebral Labeling

After segmentation, labeling of vertebral levels or discs is the second mandatory step in order to match the template to the subject's MRI (template registration).

Two vertebral levels are necessary for registering data to the template. Each of these two landmarks consists of a voxel placed in the middle of the SC, at the level of the corresponding mid-vertebral body, and assigned a relative number starting from 1 for C1 vertebra. However, SCT recently introduced the possibility to alternatively use inter-vertebral disc labels with the analogous procedure of reference-numbered voxels.

We perform this step on 3D T1w images in order to achieve better accuracy, given their higher overall quality and contrast compared to DKI ones, where vertebral discs are not clearly identifiable.

**Figure 5:**
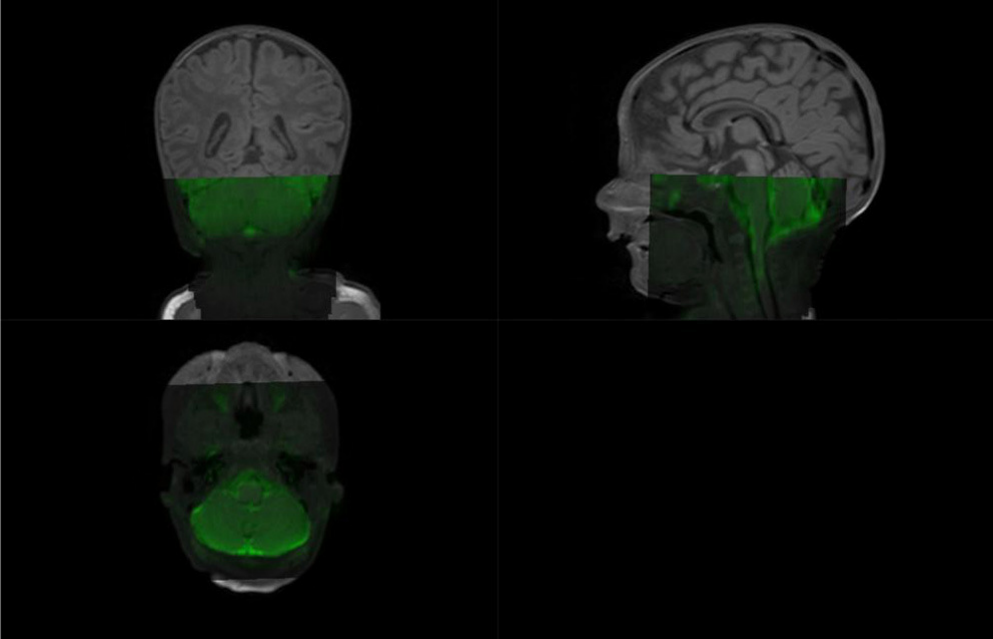
Diffusion kurtosis imaging scan overlaid on structural 3dT1w image: while both images are clearly not registered along the antero-posterior direction due to the very strong susceptibility artifact, the z-location is similar: see how the bottom tip of the cerebellum is consistent for the two scans.

Labeling from 3D T1w anatomical image is possible as it turned out to match relatively well along the superior–inferior (z) axis, the target direction of disc labeling, with the DKI scan—not along the anterior–posterior or right–left direction (see Figure 5 and, for an even clearer representation, Supplementary Figure S5).

Vertebral labeling is typically done using an automatic method *sct_label_vertebrae*, which finds the C2–C3 disc and then locates neighboring discs using similarity measure with the PAM50 template at each specific level (59).

The default SCT procedure *sct_label_vertebrae* fails in automatically detecting the C2–C3 vertebral disc once again because of the small size of spines at issue and low image contrast compared to adults.

Therefore, we manually create labels with the command *sct_label_utils* through interactive viewer option provided by SCT (flag *-create-viewer*) with little to no waste of time.

Specifically, vertebral labeling was created at the posterior tip of the top of C1 vertebra and at the C3–C4 disc, centered in the cord. Manual intervention only took a few seconds per subject (Supplementary Figure S3).

#### Registration to PAM50 Atlas

Registration between the subject's diffusion and atlas space is a very demanding task in case of neonatal imaging given the lack of a specific pediatric atlas compatible with SCT (one is currently under creation, https://github.com/neuropoly/spinalcordtoolbox/issues/2530). We thus use PAM50 atlas (60), an adult template for MRI of the full SC and brainstem in the same coordinate system, as the ICBM152 (MNI) brain template, allowing us to conduct simultaneous brain/spine studies. It consists of a T1w, T2w, T2^*^w, white and gray matter probabilistic atlas and white matter atlas of tracts as well as probabilistic labeling of spinal levels. The template has been constructed from straightened SC for facilitating the registration and visualization of results.

*sct_register_to_template* is the main command for registering one subject to the template and *vice versa* since it outputs the forward and backward warping fields. We choose the subject's native diffusion space as target of registration transforms as the straightening required by the opposite strategy would cause through-plane interpolation errors which would bias the following extraction of diffusion measures (61).

Moreover, we suggest employing T1w atlas image for its better contrast similarity with DKI scan compared to T2w.

Application of default command does not produce satisfactory results, stressing the need to tweak all the input parameters to deal with our particular contrast and resolution. Given the presence of artifacts and some inherent features (e.g., low CSF/cord contrast) that could compromise the registration, we use SC segmentation as input for the algorithm to ensure maximum robustness.

**Figure 6:**
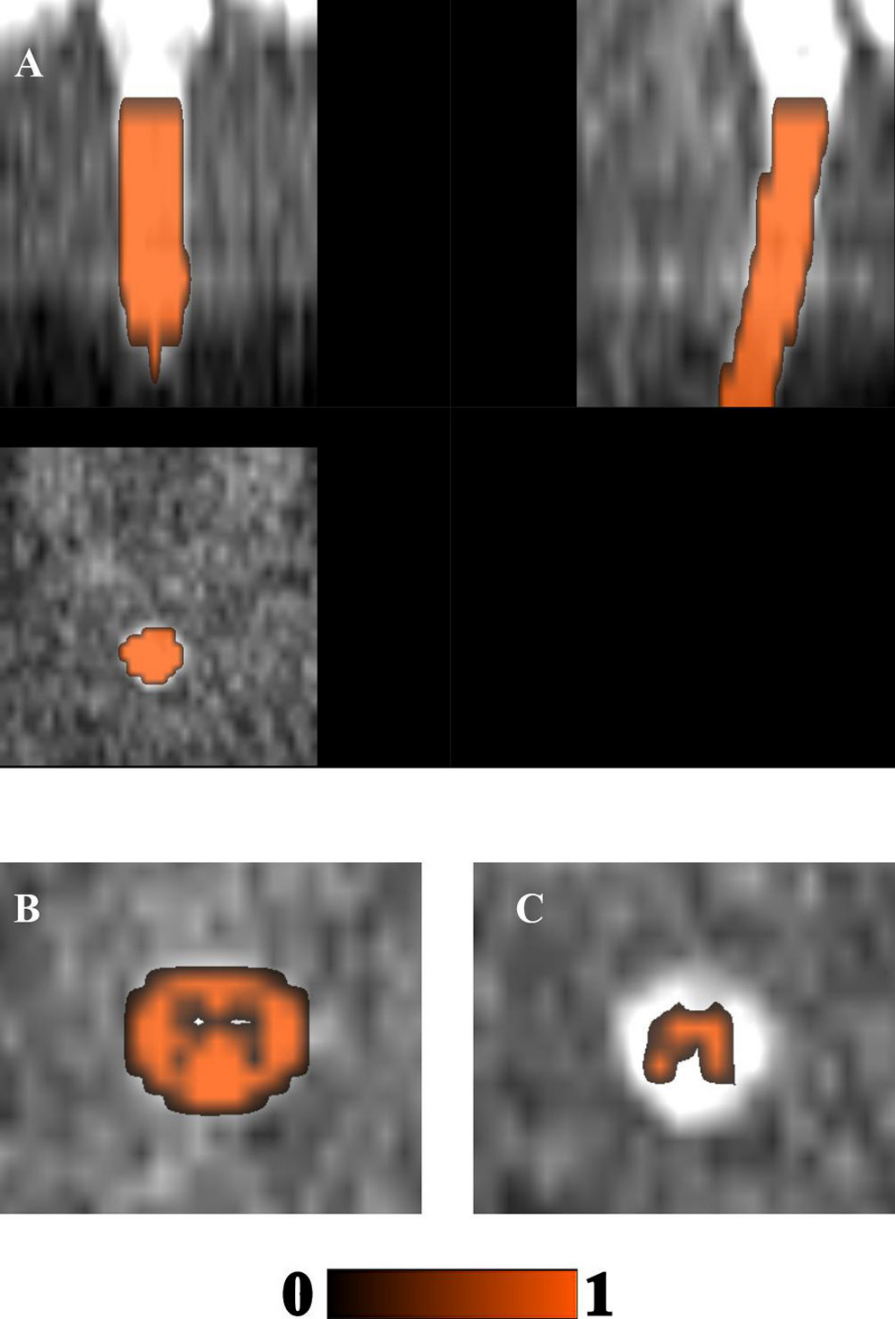
Registration with PAM50 atlas and region-of-interest detection through atlas-based approach: **(A)** PAM50 atlas' cord segmentation binary mask; **(B)** white matter; and **(C)** gray matter probabilistic masks warped to the subject's diffusion kurtosis imaging motion-corrected mean image.

Registration is then built through multiple steps by increasing the complexity of the transformation performed in each step (starting with large deformation with low degree of freedom and finishing with local adjustment). Specifically, the first step consists in vertebral alignment, that is, vertebral level matching between the subject and the template based on the posterior edge of the intervertebral discs provided by previous manual vertebral labeling. The second step is slice-wise center of mass alignment between the two images, using *centermass* algorithm instead of the default *centermassrot* (which also includes rotation alignment) because the cord is quasi-circular, and cord angle estimation is not reliable here. The third step is R–L scaling along the x-axis, followed by A–P alignment to match the segmentation borders along the y-axis, with the ultimate aim of accommodating the very small SC size. Finally, iterative slice-wise non-linear registration is performed through non-linear symmetric normalization regularized with b-splines (62) using information from the comparison of CC metric between the two images, which allows the refinement of SC shape. Once the algorithm is completed, one can assess the quality of registration through visual evaluation and inspection of the QC module and thus warp the template and all its objects to each subject's DKI image (Figure 6).

The current selection of parameters and steps successfully worked for our scans since the atlas registration algorithm robustly achieved convergence, as verified through inspection of the QC feature.

#### Computation of Diffusion Metrics

The endpoint of previous preprocessing and processing steps is computation of diffusion parametric maps, from which quantitative summary measures requested by the particular study in question were extracted. We estimate diffusion parametric maps through DIPY software (v. 1.4.0) (63).

To avoid unnecessary calculations on the background of the image, we use a mask created by dilating the spinal cord segmentation (through *sct_maths* command) because values outside the binary cord mask are important for proper accounting of the PVE, which have to be minimized in every possible way (64). Indeed this phenomenon, because of the coarse resolution of MRI with respect to SC anatomy, may make the apparent value within a boundary voxel be a mixture between the WM and CSF compartment, thus yielding an inaccurate quantification of diffusion measures.

Since the DKI model involves the estimation of a large number of parameters (65) and is more sensitive to artifacts (66), we choose to further suppress the effects of noise and artifacts before diffusion kurtosis fitting using 3D Gaussian smoothing (with a Gaussian kernel with full width at half maximum = 1.25) as suggested by pioneer DKI studies (2). This also helps in addressing the issue of implausible negative values inherent to DKI fitting (67).

The following parametric maps can thus be generated: mean diffusivity (MD), axial diffusivity (AD), radial diffusivity (RD), fractional anisotropy (FA) and mean kurtosis (MK), axial kurtosis, radial kurtosis, kurtosis fractional anisotropy, and mean signal kurtosis (MSK).

Given the low-angular-resolution data available, to ensure the robustness and reproducibility of the parameters' estimates, we opted for just computing DTI measures, whose reference tensor can be correctly estimated from at least six independent directions, and MSK. The latter is a robust scalar kurtosis index that can be estimated independently from the acquisition scheme (68, 69). Indeed fitting MSDKI is well posed without relying on the full DK tensor, which would require a minimum of 15 non-collinear directions per *b*-value. Moreover, this measure is generally more robust to low-SNR situations as in the case of neonatal imaging.

MSK can be seen as a proxy for the MK, showing to present nearly identical contrast while improving the robustness and reproducibility of the kurtosis metrics, and results in parameter maps with enhanced quality and contrast. Specifically, this measure turns out to be less sensitive to thermal noise and imaging artifacts and thus drastically reduces black voxels intrinsic to DKI and challenging the visual and statistical analysis of potentially clinically relevant biomarkers of tissue integrity. Moreover, as previously pointed out (69), standard kurtosis measures depend not only on microstructural properties but also on mesoscopic properties such as fiber dispersion or the intersection angle of crossing fibers. In contrast, MSK has the advantage of being decoupled from the confounding effects of tissue dispersion and crossing (68, 70).

Supplementary Figure S1 provides a visualization of the overall axial diffusion maps, including both DTI and MSDKI metrics, for an example subject.

### Postprocessing

Thanks to this atlas-based analysis approach, it is possible to perform a cord-specific quantification of diffusion metrics through the *sct_extract_metric* command, also restricted to specific regions of interest (ROIs; labels used by default are taken from the PAM50 template, *e*.*g*., WM tracts, flag *-l*), vertebral levels (flag *-vert*), or slice (flag *-z*), according to the specific clinical needs concerned.

Along with WM and GM probabilistic masks as a whole (Figures 6B,C), normally investigated in medical practice, one can carry out ROI detection also in specific tracts according to the clinical question (15 WM tracts and three GM regions available in total for each side).

In our example, neither DKI nor structural images ensured sufficient WM–GM–CSF contrast to perform any manual detection of ROIs in contrast to the high-contrast PSIR image of Panara et al. (45), whose acquisition time would be too long for neonates. Therefore, we exploited a good registration outcome for automatic delineation of ROIs through atlas-based approach.

We opted using lateral cortico-spinal tracts (CSTs) as ROIs for consistency with (45)—though grouping together the left and right sides in order to gain robustness by increasing the volume fraction as suggested in De Leener et al. (29)—as well as WM and GM.

We then computed the average of each diffusion measure (MD, AD, RD, FA, and MSK) across the C1–C4 vertebral levels since outside of these levels the registration is inaccurate and/or the MRI signal may be corrupted. We thus checked through the QC module if the correctly segmented slices corresponded to the same vertebral levels across subjects, starting from the first slice containing only SC (excluding cerebellum, Supplementary Figure S2C).

Moreover, estimation of DTI and MSDKI weighted average metrics was limited to those slices where the SC segmentation is accurate: outside the segmentation mask, the metrics would indeed be irrelevant. This was obtained by multiplying the segmentation mask by the specific WM, GM, and CST atlas labels. We quantified the diffusion metrics using weighted average estimation to minimize PVE and avoid bias into the resulting metrics by the surrounding tissues (e.g., CSF). This is one of the recommended methods especially in case of noisy images and small tracts as in our case. We assessed the associated voxel fraction to quantify the reliability of our diffusion measures: as demonstrated in De Leener et al. (29), having at least 240 voxels results in an error smaller than 1%, while having 30 voxels results in an error inferior to 2%. In this example, the metrics were computed based on averages of 178.3, 50.5, and 31.5 voxels in WM, GM, and CSTs, respectively, thus assuring sufficient accuracy of the estimates.

### Case Study

Periventricular WM injury is the most frequent type of brain lesion in preterm infants, and the spatial extent and location of WM injury correlate with distinct clinical outcomes, including cerebral palsy and motor impairment (71).

Given the strong association of WM injury with the motor function development of preterm neonates, we hypothesized that periventricular punctate WM lesions at TEA could be associated with regionally specific alterations in the cSC microstructure.

A similar approach was already used by Panara et al. (45) to characterize cSC microstructural abnormalities in a cohort of adult patients with previous unilateral ischemic stroke in the vascular territory of the middle cerebral artery. The DTI and DKI diffusion measures in cSC resulted to be valuable imaging markers for predicting clinical outcome. In particular, a significant reduction of FA and MK was observed in the affected lateral WM bundle of the cSC, correlating with the severity of motor dysfunction.

Accordingly, the ultimate goal of our study was to verify whether the presence of periventricular WM lesions affects the cSC tract development. Specifically, we aimed to compare the DTI and MSDKI measures of cSC in two groups of preterm neonates: (i) with punctate periventricular white matter lesions and (ii) with normal brain MRI (controls).

## Results

### Population Size and Classification

In order to investigate clinical differences among the acquired subjects, we grouped the infants as follows: (i) 9 subjects with punctate PWMI and (ii) 9 subjects with normal brain MRI, used as the control group.

At the QC phase, in accordance with the expert neuroradiologist, we opted to exclude one control subject due to excessively poor image quality (*i*.*e*., signal leakage at the C1–C3 level; Supplementary Figures S2A,B).

Therefore, the final number of subjects under analysis was 9 and 8 infants for the patient and the control groups, respectively.

### The Role of Denoising

As mentioned above, neonatal imaging is inherently affected by low SNR and sensitivity to imaging artifacts. Proper denoising of scans is therefore a crucial step in the processing pipeline. Above all, we thus focused on quantitatively assessing the contribution of Patch2Self denoiser on subsequent analysis.

Firstly, we computed the average SNR on *b* = 0, *b* = 700, and *b* = 2,100 images for all subjects and across all slices belonging to the C1–C4 district of our interest. For this task, we resorted to SCT function sct_compute_snr. The latter exploits the methods described in Dietrich et al. (72).

Specifically, we have taken into account the spatially varying and parameter-dependent nature of noise distribution in case of parallel imaging by choosing the so-called *mult* method. According to this definition, the noise of a single voxel is described by the stochastic variation of its signal intensity in repeated acquisitions.

Since this approach has the weakest requirements on the statistical and spatial distribution of noise, it turns out to be valid also in the case of increasingly complex MRI systems (e.g., multiple channels and complex reconstruction algorithms), and it is thus used as the standard of reference with which to compare the validity of other existing methods. In the absence of back-to-back scans with the same parameters (to use the default “diff” method), we looked at the “*mult*“ approach as the best option possible for our kind of input data.

**Figure 7:**
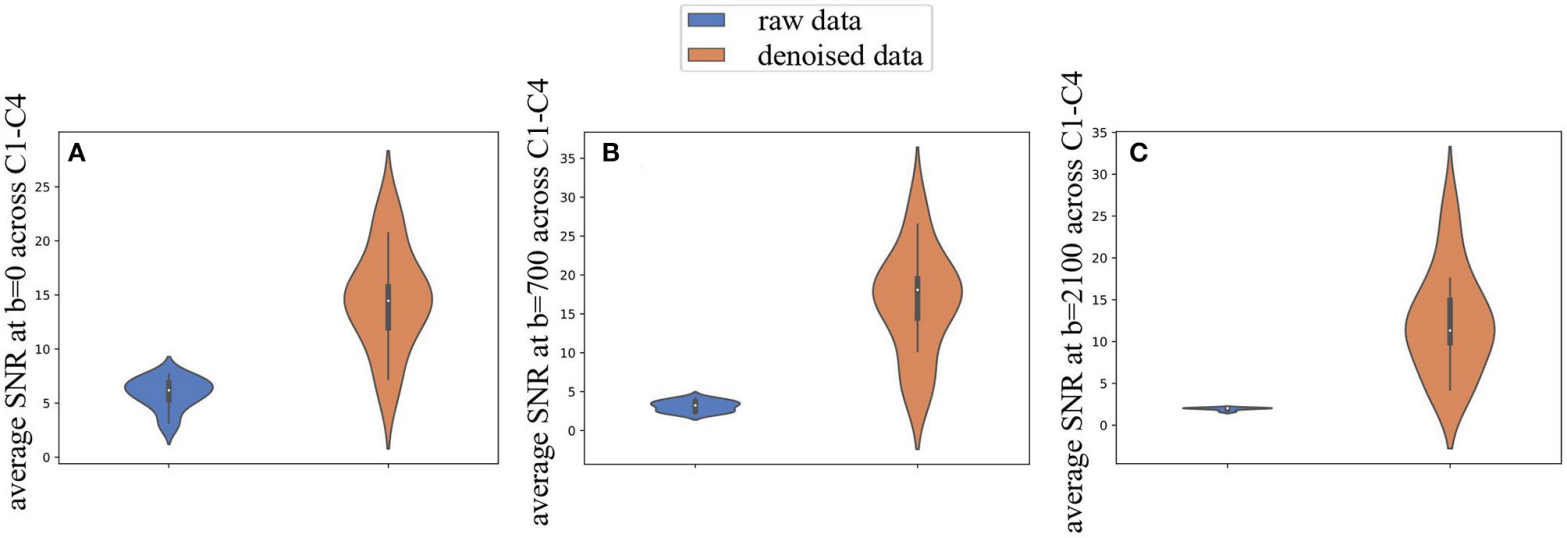
Effects of Patch2Self denoising on noise at different diffusion weightings. **(A)** Average signal-to-noise ratio computed on *b* = 0 images, **(B)** *b* = 700, and **(C)** *b* = 2,100 s/mm^2^ increases in all the cohort, across C1–C4 vertebral levels under analysis, when including denoising with Patch2Self algorithm in the processing pipeline.

We found an increase in mean SNR after applying Patch2Self at *b* = 0 s/mm^2^ (5.88 ± 1.41 vs. 14.64 ± 4.53), *b* = 700 s/mm^2^ (3.12 ± 0.67 vs. 16.47 ± 6.62), and *b* = 2,100 s/mm^2^ (1.95 ± 0.16 vs. 12.31 ± 6.20). Hence, this evidence subsists not only for *b* = 0 images, agnostic from signal attenuation related to diffusion and thus exhibiting the highest SNR, but also for non-*b* = 0 shells (Figure 7).

We then inspected the impact of denoising on microstructure model fitting, a critical step often leading to degeneration of parameter estimates due to the low SNR of dMRI acquisitions.

**Figure 8:**
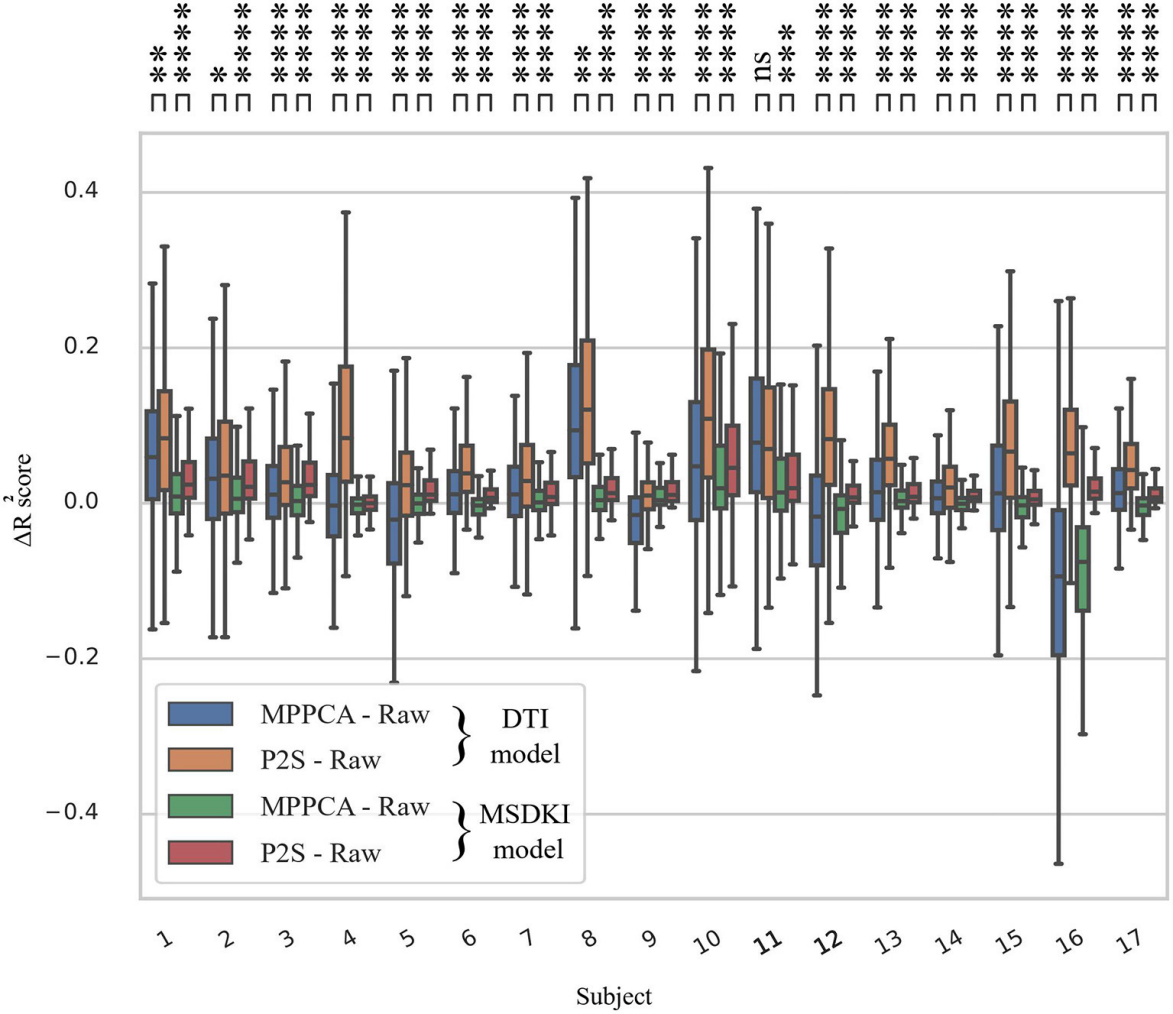
Box plots quantifying the increase in *R*^2^ metric after fitting downstream the diffusion tensor imaging and MSDKI models for the whole spinal cord volume across all subjects. The *R*^2^ improvements in each case are plotted by subtracting the scores of model fitting on undenoised data (raw) from *R*^2^ of fitting each denoised output. Note that the consistency of microstructure model fitting on Patch2Self (P2S) denoised data is higher than that obtained from Marchenko–Pastur, especially as regards MSDKI model. Ns, 5.00e−02 < *p* ≤ 1.00e + 00; *, 1.00e−02 < *p* ≤ 5.00e−02; **, 1.00e−03 < *p* ≤ 1.00e−02; ***, 1.00e−04 < *p* ≤ 1.00e−03; ****, *p* ≤ 1.00e−04 in two-sided *t*-test with Bonferroni correction.

Specifically, we applied the DTI and MSDKI models on raw and denoised data, resorting both to traditional Marchenko–Pastur PCA (MP-PCA) (73) and to Patch2Self method. We opted to compare our denoising procedure with MP-PCA since it represents the current state-of-the-art unsupervised method for denoising DWI. MP-PCA exploits the redundancy in multidirectional dMRI data by identifying the noise-only principal components using the knowledge that the corresponding eigenvalues are described by the universal MP distribution, parameterized by the noise level. In order to compare the goodness of each fit, we performed a *k*-fold cross-validation (*k* = 2) (74) across the whole volume of masked data for all the datasets at disposal. As a standard measure for quantifying the goodness of fit in linear regression models, we computed the coefficient of determination (*R*^2^ score = $1-\frac{\sum_{i}(y_{i}-f_{i}){\;}^{2}}{\sum_{i}(y_{i}-y){\;}^{2}}\;$, with *y*_1_…*y*_n_ ≜ observed values, *y* ≜ mean of observed values, and *f*_1_…*f*_n_ ≜ fitted values). In Figure 8, we depict the improvement of the *R*^2^ metric by simply subtracting the *R*^2^ scores of fitting undenoised data from Marchenko–Pastur and Patch2Self denoised data for both the DTI and MSDKI models. We could observe a consistent trend across all subjects: Δ*R*^2^ shows a significant increase from MP-PCA to Patch2Self method for all the cohorts in case of fitting the MSDKI model and for all subjects except for one in case of the DTI model (two-sided *t*-test with Bonferroni correction).

Our observation suggests that Patch2Self proves to be particularly suitable for the DKI model.

### MSK Decreases in Patients With PWMI Lesions

**Figure 9:**
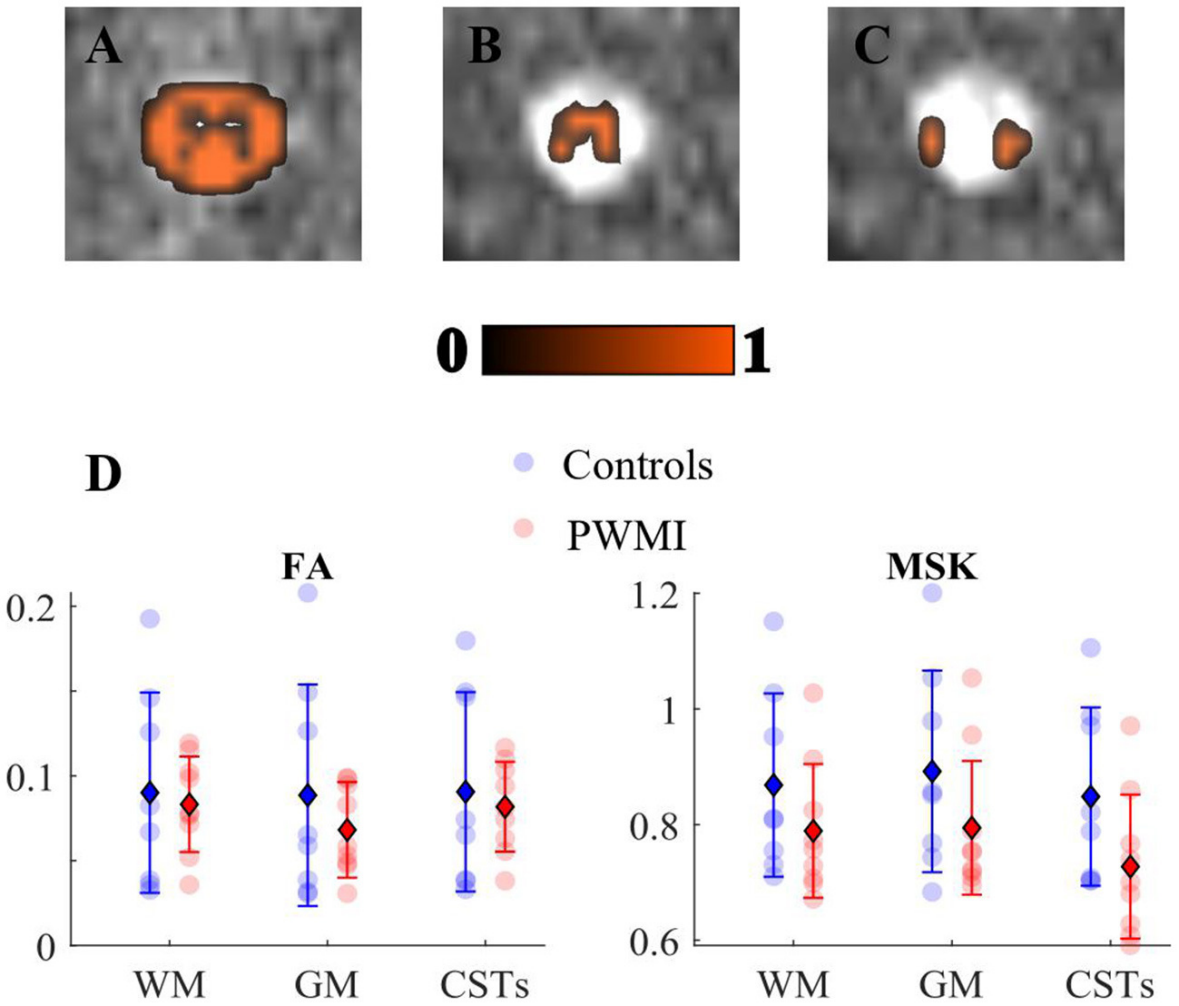
Mean signal kurtosis (MSK) decreases in neonatal periventricular white matter injuries: **(A)** white matter, **(B)** gray matter, and **(C)** cortico-spinal tract regions of interest (ROIs) overlaid on diffusion kurtosis imaging motion-corrected image. **(D)** Scatter plots of fractional anisotropy (FA) and MSK in group subjects across the aforementioned ROIs: colored spots indicate single subject's value for each metric; as reported in the legend, controls' measures are in blue, whereas the periventricular white matter injury group is in red. The units for MSK are in mm^2^/s, while FA is dimensionless. Error bars displaying mean (diamond) and standard deviation (bars) are overlaid on scatter plots.

In an initial evaluation based on the limited sample size available, we detected an increase in MD, AD, and RD (Supplementary Figure S4), parallel to an overall decrease in FA and MSK (Figure 9) in preterm neonates with PWMI (18–21, 24, 75–78).

This decrease was more pronounced in MSK than FA (Figure 9).

This visual trend of diffusion measures has been supported by a statistical survey, which is to be considered as preliminary given the very low sample size. We thus performed Scheirer Ray Hare Test, that is, the non-parametric alternative of 2-way ANOVA, to assess the presence of statistically significant differences in DTI- and MSDKI-derived metrics between the patient and control groups. Specifically, we analyzed the effect of diagnosis (PVWMI/control) and ROI (WM/GM/CSTs) on each diffusion measure **(**MSK, FA, MD, AD, and RD**)**. We showed that there is no statistically significant interaction between the effects of diagnosis and ROI for any of the DTI and MSDKI measures (Supplementary Table S1). Similarly, the simple main effects analysis showed that ROI does not significantly affect any diffusion parameter. Conversely, the simple main effects analysis indicated a statistically significant effect of diagnosis exclusively on MSK, regardless of the ROI examined (*p* = 0.0153).

Then, we wanted to assess if the MSK and FA means were significantly different between the two groups in the different ROIs. We thus conducted Mann–Whitney *U*-test between each patient/control pair for each ROI (GM, WM, and CSTs) separately for MSK and FA. Given the generally low power of the statistical tests due to the limited number of subjects, we decided to quantify the common language effect size given its independence from sample size. In this case, we also reported a non-significant (*p* > 0.05) difference of both MSK and FA values between the two groups in all ROIs.

Nevertheless, we observed that MSK in CSTs exhibits the combined lowest *p*-value (*p* = 0.067, uncorrected) and the highest effect size (0.77), corroborating the observed decrease of MSK in the patient group with respect to the controls (Figure 9).

## Discussion

### Research Question

In the present study, we tested the pipeline of SC DKI analysis in a group of neonates with PWMI, a form of mild WM injury frequently diagnosed in preterm infants. PWMIs are seen at brain MRI as small, focal, multiple alterations of signal intensity (high on T1 and/or low on T2) in periventricular WM. The long-term outcome of neonates with PWMI seems to be related to the number of lesions, their pattern, and their localization. Notably, several studies have shown that a greater lesion load of PWM and the involvement of frontal WM are associated with a higher risk of adverse neurodevelopmental outcome, affecting both motor and cognitive functions (79). Moreover, periventricular WM lesions in preterm neonates are associated with region-specific changes in MD, FA, RD, and AD in several cerebral WM tracts that might explain the abnormal development of long-term neurological functions (80). Specifically, the involvement of pyramidal tract fibers in the periventricular WM has been demonstrated to be a relevant factor for motor dysfunction in children with PWMI (81). In our study, we found that microstructural changes can be detected by using an advanced DKI analysis also in the GM and WM of cSC of preterm neonates with PWMI studied at a term-equivalent age, thus suggesting that DKI parameters could be used as markers to unravel underlying subtle microstructural lesions. Moreover, our preliminary findings confirm the hypothesis that, in preterm neonates with PWMI, WM microstructure alterations extend beyond the immediate area of periventricular injury, widening distally also in the cSC (82, 83).

Furthermore, the range of values of DTI measures is consistent with normative values on healthy pediatric SC (24). The MD, AD, and RD values are higher, while the FA values are lower compared to equivalent measures on older cohorts of patients (i.e., children/adolescents) (18–21). This may be partially due to the sensitivity of FA to denoising, which can imply a reduction in this metric. Moreover, this trend is in line with the simultaneous age-related decrease in MD, AD, and RD and increase in FA metrics reflecting progressive maturation, myelination, and fiber packing and thickening within the SC, similar to that observed in the brain (77, 78).

Conversely, the definition of a normative variation of DKI measures across ages from newborns to adults will be feasible after further investigations from early stages of development.

Further analyses on a wider cohort of neonates are necessary to confirm these preliminary results and specifically to prove if the microstructural changes in cSC in preterm neonates with PWMI correlate with long-term neurological outcomes.

### Study Significance

Here we present the first application of DKI to neonatal SC through a pipeline able to perform complete processing on a subject within a clinically acceptable time (10 min on average with the current setup). As regards acquisition setting, we were able to perform a time-consuming technique like DKI using a short diffusion sequence which minimizes patient's physiological motion and which likely reflects a standard clinical scenario devoid of the latest technologies in terms of acquisition sequence optimization.

Among existing denoising strategies *via* magnitude data, thanks to its weak assumption about noise properties to be suppressed, Patch2self showed optimal performance in effectively minimizing the detrimental bias introduced by Rician noise at higher *b*-values. In turn, this minimization reduces error estimates in tensors computation and subsequently derived metrics. However, to properly break the Rician noise floor, one would need complex valued data (84) which requires specific settings during acquisitions and thus will be considered in future studies.

With regard to image processing, we opted for creating this pipeline using SCT since it represents the only existing comprehensive, free, and open-source software dedicated to the processing and analysis of SC multi-parametric MRI data. Adaptation of each image processing tools already in use for adult subjects through appropriate tuning of parameters turned out to be feasible. Indeed it allowed to successfully overcome all the issues mentioned in the “Introduction” section inherent to imaging of SC and exacerbated in case of neonatal setting, even for the most challenging steps like segmentation or registration to atlas. We were thus able to quantify diffusion measures within specific ROIs using an atlas-based approach which presents undisputed advantages compared to the usual manual drawing of ROIs. It is automatic and thus highly reproducible, it is not biased by the user's experience and knowledge of the anatomy, it is much faster than the long and tedious manual delineation of ROIs, and it allows to account for PVE.

### Added Value of DKI

The results about the feasibility of DTI and MSDKI analysis in neonatal SC subjects collected so far are preliminary but promising and demonstrate the clinical utility of combining DTI and DKI in the characterization of spinal cord pathologies.

FA reduction parallel to MD increase in patients is an expected finding consistent with existing literature and attributable to the degeneration of the diffusion barrier and loss of diffusion directionality.

Our results suggest that, although yet underused in clinical studies, MSK metrics might have an increased sensitivity in capturing alterations related to pathology, also far from the lesion site.

Such findings once again stress the importance of combining DTI and DKI metrics as complementary sensitive biomarkers in order to fully exploit the potential of dMRI compared to conventional MRI.

Our results further hint that the presence of a WM lesion in the brain might cause subsequent alterations not only in cSC WM but also in GM, as evidenced by the strong association between the brain and spine. In this respect, resorting to DKI measures becomes of utmost importance given the kurtosis sensitivity to structural changes in isotropic tissues such as GM. Indeed the range of variability of MSK metric from controls to PWMI was overall higher in GM than that of corresponding DTI measures, and a considerable decrease in case of PWMI was registered also in GM, unlike for DTI-related parameters.

If FA and, more importantly, MSK measures appeared to be more sensitive to microstructural changes related to pathology both in WM and GM, the same did not apply for WM/GM tissue differentiation.

Indeed MD, AD, and RD showed a lower value in GM with respect to WM (including CSTs) for both the control and patient groups. Conversely, FA and MSK kept comparable values for the two tissues.

Since no other studies yet exist about dMRI on this anatomical district in such age range, we had no ground-truth to compare our findings with. However, we hypothesized that, in this cohort of early preterm subjects, WM and GM already differ in terms of the amount of diffusivity but yet still not of microstructural organization or complexity.

Indeed a higher density of cell nuclei in GM than in WM translates in a decreased amount of diffusivity along all directions (mean, axial, and radial). In GM, the presence of a cell body contributes to create voluminosity in the environment, which turns into a more restricted diffusion pattern.

On the contrary, a parameter like FA, tightly dependent on GA and strongly modulated by myelin growth, may not be yet particularly sensitive to the different microstructure between the WM and GM portions. The same happens for MSK, index of microstructural complexity, related to brain maturation, and supposedly not so different in SC areas at this early stage.

In any case, a more comprehensive corroboration and explanation of our results is expected after collecting an adequate number of subjects to carry out a robust statistical survey. An in-depth interpretation of the single metrics is out of the scope of this paper. Here we just dwell on exploring the comparison with the work on adults which served as a starting point. Indeed our statistical findings may be strongly affected by the limited sample at disposal, making the power of the tests too low to detect meaningful differences in the data. That is precisely why the conclusions drawn are just preliminary and mainly based on observations of the scatter plots of the present data. It is therefore once again made clear that this is about a pilot study that will help design a future well-powered study able to provide valid and generalizable conclusions.

### Comparison With Adult Study

Since MSK has proven to be a good approximation of MK, here we assimilated it with standard the DKI metric used in Panara et al. (45). The trend of both FA and MSK agreed with the aforementioned study. Specifically, they both exhibited a reduction in WM, GM, and CSTs in case of pathology (Figure 9).

Since FA is known to be an index of structural integrity (85) and MSK is a marker of tissue microstructure complexity (2), our findings suggest that, in case of an overlying WM brain lesion, a loss of integrity and complexity is registered also in SC WM tracts below, with a more isotropic diffusion pattern due to the disruption of WM tracts. MD, AD, and RD (Supplementary Figure S4) also followed the same trend, with an increase in the case of lesioned subjects as in Panara et al. (45). Hence the hypothesis that the microstructural impairment of SC could be related to distant lesions of cerebral WM, already verified for adults with stroke lesions, would also subsist in infants with smaller prematurity-related WM lesions.

### Study Limitations

The present pipeline has been designed to fulfill specific requirements such as short acquisition time and minimal modifications to the routine protocol in use at the hosting center. As a result, any improvement in the acquisition setup of our pipeline will bring forth even stronger and more comprehensive results.

The major limitations of this procedure consist in basing on an adult atlas, where the exact location of tracts may not perfectly correspond to the neonatal images despite the good adjustment of registration parameters. Unfortunately, to the best of our knowledge, a comprehensive freely available SC atlas for this age range does not exist yet. The current pipeline will definitely benefit from the introduction of a pediatric atlas into SCT. A valid alternative strategy could also be represented by inserting into the clinical routine protocol multi-echo FFE, a T2^*^-weighted spoiled gradient echo sequence considered a fast alternative to PSIR although useful for displaying the internal architecture of the spinal cord and for performing manual detection of ROIs while waiting for a neonatal-specific atlas.

Another limitation is the comparison of DTI and DKI metrics from the same acquisition protocol. On the one hand, indeed DTI would settle for a much simpler, single *b*-value protocol in favor of shorter TE, less susceptibility artifacts, and higher SNR, also allowing for cardiac gating and motion compensation techniques. On the other hand, the present study has been specifically conceived to develop and test a methodological pipeline for conducting DKI-related studies in neonatal SC data. Therefore, our study required a HARDI protocol rather than an acquisition setting optimized for DTI. The same approach was used in the reference corresponding adult survey, wherein the authors resort to the same acquisition protocol to estimate both DTI and DKI metrics. Furthermore, this decision was supported by two further studies. First, it has been demonstrated that the *b*-value dependency of the DTI model—which hampers the interpretation and comparison of various diffusion tensor imaging studies—can be partially reduced by fitting the model to DWIs acquired with multiple non-zero *b*-values, even though not to the degree obtained with the DKI model (86). Finally, given the high dependence of DTI-derived scalar indexes on acquisition parameters (87), we considered it more appropriate to acquire both DTI and DKI measures of interest under the same condition in order to compare them as consistently as possible.

Moreover, the image quality could be further improved: the scans we acquired are extracted from routine clinical protocol and consequently prone to noise and artifacts due to the short acquisition time dictated by clinical needs and to the lack of specific, spatially selective MR sequences.

Starting from more advanced hardware tools may significantly increase the image quality and thus accuracy of the estimated metrics. Resorting to optimized acquisition sequences would also allow to increase the resolution of HARDI acquisition scheme and thus to exploit all standard DKI measures, which can, in turn, increase the amount of diagnostic information. Partially borrowed from the reference adult study, our acquisition protocol certainly has room for improvement—for instance, rather than prioritizing the voxel size, reducing TE in favor of higher SNR and better contrast could be an option.

We acknowledge the protocol in use to be on the edge for HARDI schemes required by DKI. However, this represents a first attempt to customize an advanced dMRI acquisition setting within a clinical routine protocol, already long in itself since made up of multiple MRI sequences in order to increase the diagnostic possibilities. Nevertheless, we appropriately addressed this issue at the DKI tensor and measures computation phase to ensure reliability and accuracy in their estimates. As already mentioned, a valuable alternative would be resorting to fast DKI methods, particularly suitable for imaging of neonates, thanks to their inherent time reduction.

### Future Developments

Validation of the current pipeline can be made by testing it to a larger cohort of subjects, possibly investigating lower SC tracts also, including thoracic and lumbar districts, and extending the studies to different clinical cases, preferably focusing on a determined pathology—for example, it would be interesting to explore the long-term correlations between DKI measures and specific clinical scores as done in Panara et al. (45), where diffusion measures have been related to motor performance indexes.

A further step may be adapting this analysis pipeline to other promising higher-order diffusion models requiring multi-shell acquisition such as NODDI (88).

## Conclusion

In this work, we have shown how accurate adjustment and parameters' tuning of processing algorithms customized for adult SC opens up new horizons in exploiting the increased ability of advanced dMRI models, also in neonatal domain, where they had never been utilized before.

Indeed even starting from low-quality data acquired for diagnostic purposes and thus suboptimal, we were able to extract from DKI information which were relevant for diagnosis.

The case study proposed in this paper is just an example of the potential relapses of this semi-automated pipeline, which paves the wave for applying advanced dMRI models to the neonatal setting in a wide range of potential clinical applications. In particular, the possibility of successfully exploiting the increased sensitivity and sensibility inherent to a DKI methodology also into the neonatal setting would indeed be extremely useful for throwing light on complex diseases related to this critical phase of development and to deepen the knowledge about the relationship between the brain and the SC at birth.

## Data Availability Statement

Raw diffusion data used in this study is not able to be made openly available due to privacy restrictions of clinical data imposed by the Gaslini Hospital's administration. Regarding code availability, a specific pipeline compatible with SCT is integrated in an open-access GitHub repository within “SCT-pipeline”, a site gathering various pipelines compatible with SCT for processing MRI data (https://github.com/sct-pipeline/pediatric-genova).

## Ethics Statement

The studies involving human participants were reviewed and approved by Comitato Etico Regione Liguria. Written informed consent to participate in this study was provided by the participants' legal guardian/next of kin. Written informed consent was obtained from the minor(s)' legal guardian/next of kin for the publication of any potentially identifiable images or data included in this article.

## Author Contributions

RT contributed to conceptualization, methodology, software, writing—original draft, and visualization. MR contributed to conceptualization and supervision. DT contributed to conceptualization, validation, investigation, resources, and supervision. MS and AR contributed to validation, investigation, resources, and supervision. JC-A contributed to writing—reviewing and editing and supervision. MF and GA contributed to conceptualization, writing—reviewing and editing, and supervision. All authors contributed to the article and approved the submitted version.

## Conflict of Interest

The authors declare that the research was conducted in the absence of any commercial or financial relationships that could be construed as a potential conflict of interest.

## Publisher's Note

All claims expressed in this article are solely those of the authors and do not necessarily represent those of their affiliated organizations, or those of the publisher, the editors and the reviewers. Any product that may be evaluated in this article, or claim that may be made by its manufacturer, is not guaranteed or endorsed by the publisher.
