# Supplementary material for: Diffusion Kurtosis Imaging of Neonatal Spinal Cord in Clinical Routine
Source: Front Radiol. 2022 May 23;2:794981. doi: 10.3389/fradi.2022.794981 (PMC10365122; doi:10.3389/fradi.2022.794981)
Supplement: Supplementary file 1 [file Data_Sheet_1.docx]

Supplementary Material

# Supplementary Figures

**
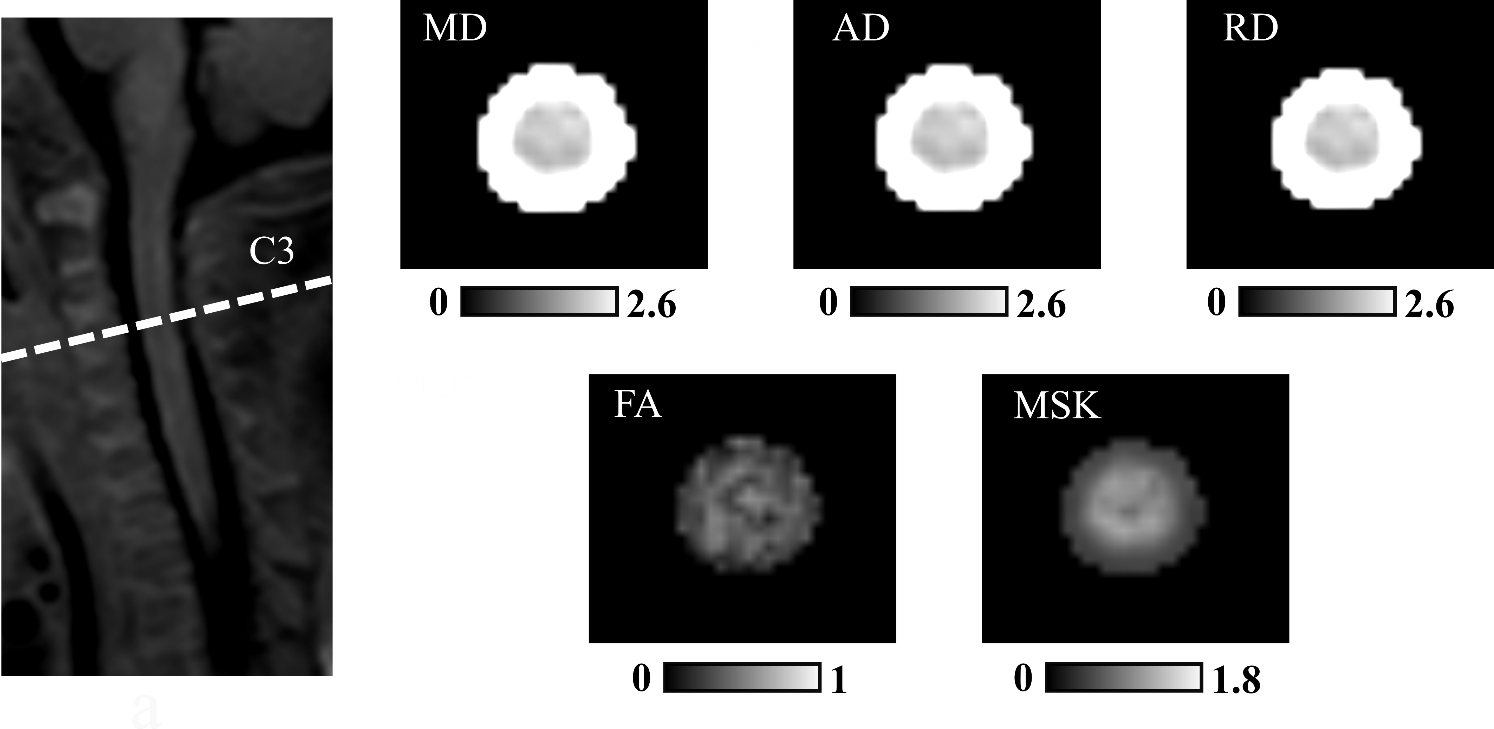
Figure 1S.** **Diffusion and kurtosis maps at the mid-C3 level for one example subject:** Units for MD, AD and RD are µm^2^/s, for MSK mm^2^/s, while FA is dimensionless.


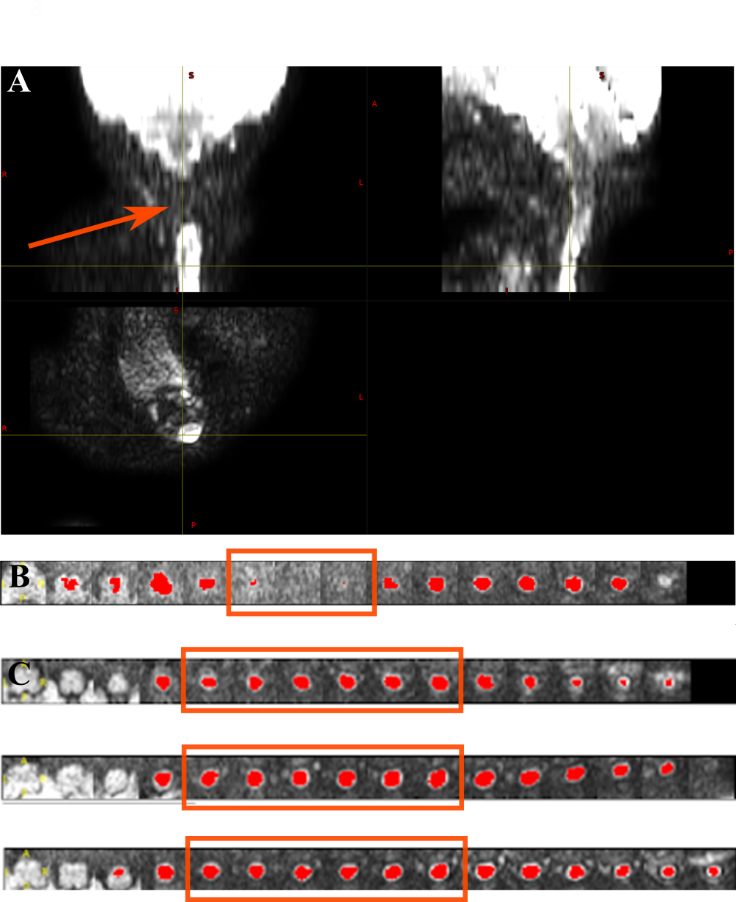


**Figure 2S.** **Quality control:** **(A)** Example of excluded DKI scan and **(B)** relative SC segmentation show signal loss across multiple slices as the coronal plane is not overlapping with the cord (ie: lordosis); **(C)** QC of C1-C4 levels: axial slices under analysis correspond to the same cervical levels for all subjects as shown in three example subjects.

**
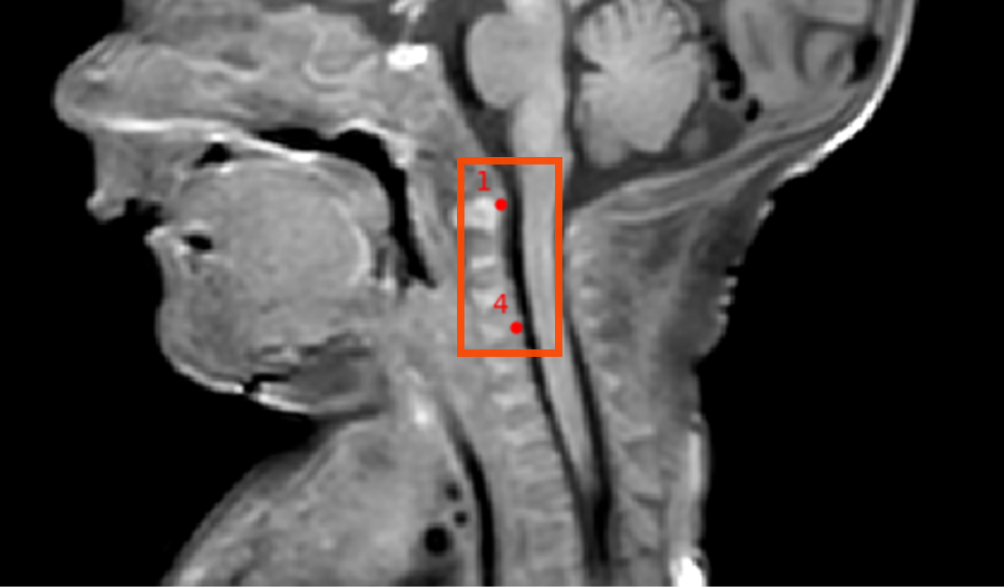
**

**Figure 3S. Vertebral labeling:** Manual labeling of top of C1 vertebra and C3-C4 disc from graphical user interface integrated in SCT.


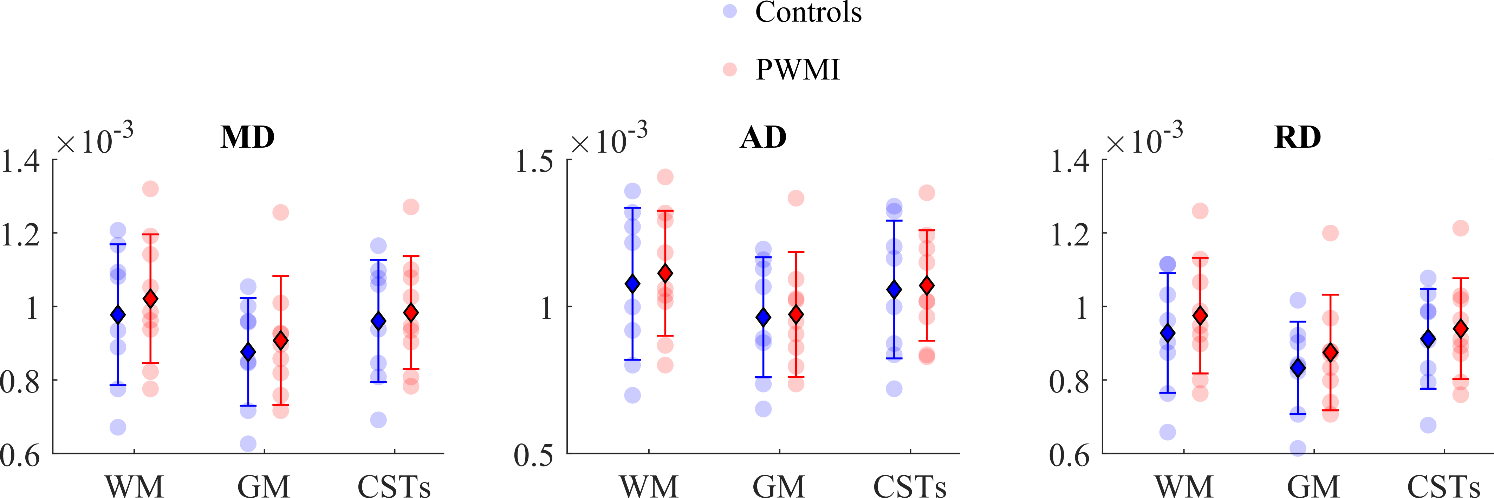


**Figure 4S.** **Extraction of diffusion measures within specific ROIs:** Scatter plots of DTI in group subjects across aforementioned ROIs: coloured spots indicate single subject’s value for each metric; as reported in the legend, controls’ measures are in blue, whereas Periventricular White Matter Injury (PWMI) group’s in red. Units for MD, AD, RD are in mm^2^/s. Error bars displaying mean (diamond) and standard deviation (bars) are overlaid on scatter plots.


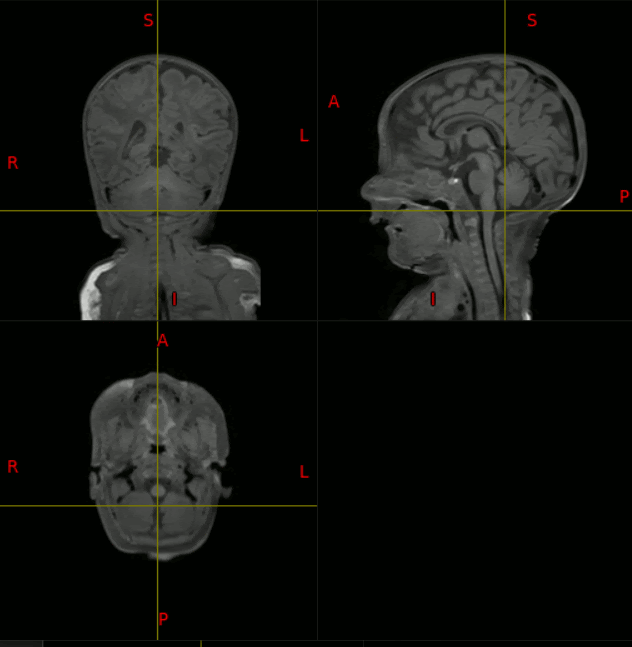


**Figure 5S.** **DKI scan overlaid on structural 3dT1w image in animated picture:** while both images are clearly not registered along the antero-posterior direction due to the very strong susceptibility artefact, the z-location is similar: see how the bottom tip of the cerebellum is consistent for the two scans

# Supplementary Tables

| **Test of Between-Subjects Effects** | | | | | |
| --- | --- | --- | --- | --- | --- |
|  | **Factor** | **SS** | **DF** | **H** | **p-value** |
| **MSK** | **diagnosis** | 1299.6 | 1 | 5.8812 | **0.015303** |
|  | **ROI** | 402.71 | 2 | 1.8224 | 0.40203 |
|  | **diagnosis*ROI** | 46.229 | 2 | 0.20921 | 0.90068 |
|  | **residuals** | 9300 | 45 | NaN | NaN |
| **FA** | **diagnosis** | 0.019676 | 1 | 8.9043e-05 | 0.99247 |
|  | **ROI** | 194.94 | 2 | 0.88221 | 0.64333 |
|  | **diagnosis*ROI** | 56.327 | 2 | 0.25491 | 0.88033 |
|  | **residuals** | 10797 | 45 | NaN | NaN |
| **MD** | **diagnosis** | 33.075 | 1 | 0.14968 | 0.69884 |
|  | **ROI** | 874.94 | 2 | 3.9595 | 0.1381 |
|  | **diagnosis*ROI** | 19.05 | 2 | 0.086209 | 0.95781 |
|  | **residuals** | 10121 | 45 | NaN | NaN |
| **AD** | **diagnosis** | 12.297 | 1 | 0.055652 | 0.8135 |
|  | **ROI** | 627.29 | 2 | 2.8388 | 0.24186 |
|  | **diagnosis*ROI** | 12.197 | 2 | 0.055196 | 0.9728 |
|  | **residuals** | 10397 | 45 | NaN | NaN |
| **RD** | **diagnosis** | 68.492 | 1 | 0.30996 | 0.5777 |
|  | **ROI** | 1043.3 | 2 | 4.7214 | 0.094353 |
|  | **diagnosis*ROI** | 16.863 | 2 | 0.076315 | 0.96256 |
|  | **residuals** | 9919.9 | 45 | NaN | NaN |
| **SS=Sum of Squares; DF=Degrees of Freedom; H= Test Statistics** | | | | | |

**Supplementary Table 1:** **two-way non-parametric Scheirer-Ray-Hare** output to assess the presence of statistically significant differences in DTI- and MSDKI- derived metrics between patient and control groups.

| **Multiple Comparisons of means** | | | | | |
| --- | --- | --- | --- | --- | --- |
|  | **ROI** | **U-val** | **RBC** | **CLES** | **p-value** |
| **MSK** | **WM** | 48.5 | -0.347222 | 0.673611 | 0.247923 |
|  | **GM** | 49.5 | -0.375 | 0.6875 | 0.210682 |
|  | **CSTs** | 55.5 | -0.541667 | **0.770833** | **0.06734** |
| **FA** | **WM** | 35.5 | 0.013889 | 0.493056 | 1 |
|  | **GM** | 39.5 | -0.097222 | 0.548611 | 0.772694 |
|  | **CSTs** | 34.5 | 0.041667 | 0.479167 | 0.923295 |
| **ROI=Region of Interest; WM=White Matter; GM= Gray Matter; U-val=U-value; RBC=Rank-Biserial Correlation; CLES= Common Language Effect Size** | | | | | |

**Supplementary Table 2:** **Mann-Whitney U Test** (Wilcoxon rank-sum test) as non-parametric version of the independent T-test for pairwise post-hoc comparisons between patient and control group within each ROI, limited to MSK and FA - the two variables of interest for this study.
